# Real Time Monitoring of Inhibition of Adipogenesis and Angiogenesis by (−)-Epigallocatechin-3-Gallate in 3T3-L1 Adipocytes and Human Umbilical Vein Endothelial Cells

**DOI:** 10.3390/nu7105437

**Published:** 2015-10-27

**Authors:** Wenjing Tang, Huanlei Song, Wei Cai, Xiuhua Shen

**Affiliations:** 1 Department of Clinical Nutrition, Xin Hua Hospital, School of Medicine, Shanghai Jiao Tong University, Shanghai 200092, China; twj_2008@163.com (W.T.); song090928@sina.com (H.S.); 2 Department of Nutrition, School of Medicine, Shanghai Jiao Tong University, Shanghai 200025, China

**Keywords:** (−)-Epigallocatechin-3-gallate (EGCG), vascular endothelial growth factor (VEGF), 3T3-L1 cells, angiogenesis

## Abstract

Little is known about the effect of (−)-epigallocatechin-3-gallate (EGCG) on angiogenesis in adipocytes. We aimed to test the effect of EGCG on the expression of vascular endothelial growth factor (VEGF) in adipocytes. The levels of VEGF secretion, the expression of VEGF message ribonucleic acid (mRNA) and VEGF protein in 3T3-L1 cells were measured by enzyme linked immunosorbent assay (ELISA), real time polymerase chain reaction (PCR), and immunofluorescence staining, respectively. The xCELLigence real time cell analysis system was used to study the growth and differentiation of 3T3-L1 preadipocytes. A coculture system was used to test the effects of 3T3-L1 cells on proliferation of human umbilical vein endothelial cells (HUVECs). The conditioned media derived from 3T3-L1 cells treated with or without EGCG was used to culture the HUVECs for a tube formation assay. Peroxisome proliferator-activated receptor γ (PPARγ) and CCAAT/enhancer binding protein α (C/EBPα), two transcription factors related to both adipogenesis and angiogenesis, were examined to explore the potential mechanism. We found that all the three measurements of VEGF expression in adipocytes (mRNA, protein and secretion in media) were reduced after EGCG treatment. The growth of HUVECs co-cultured with 3T3-L1 cells was significantly increased and the conditioned media from EGCG treated 3T3-L1 adipocytes inhibited tube formation in HUVECs. Both PPARγ and C/EBPα expression in adipocytes were decreased with EGCG treatment. In conclusion, findings from this study suggest that EGCG may inhibit angiogenesis by regulating VEGF expression and secretion in adipocytes.

## 1. Introduction

Adipose tissue is characterized by its lifelong proliferation and relatively unlimited expansion. Development and maintenance of fat depots require angiogenesis, or the formation of new blood vessels, to enable delivery of oxygen and nutrients [1]. Previous evidence has shown that modulators of angiogenesis affect the expansion and metabolism of fat masses by regulating the growth and remodeling of adipose tissue vasculature [2]. Thus, regulation of adipose tissue angiogenesis may be a new preventive and/or therapeutic strategy for obesity [2,3,4].

Increasing evidence shows that adipose tissue does not simply store energy. It is also an essential endocrine organ that secretes various angiogenic growth factors to promote angiogenesis during its expansion. Examples of the angiogenic growth factors are vascular endothelial growth factor (VEGF), fibroblast growth factor (FGF), placental growth factor (PlGF) and leptin [1,5,6,7].

VEGF is critical for maintaining the viability and metabolic/endocrine functions of adipocytes because of its role in regulating vascularization and blood perfusion [8]. It has been reported that VEGF is highly expressed in murine visceral fat, and the circulating VEGF concentration is positively correlated with visceral fat volume in obese mice [9,10]. Additionally, impaired angiogenesis when caused by either VEGF-A blockade or macrophage depletion in postnatal mice interferes with adipose tissue development [11]. Furthermore, the elevated VEGF levels and the visceral fat accumulation in obese human subjects were reduced after a body weight decrease [12]. These studies indicate that VEGF is essential for adipocyte survival, and that targeting VEGF in adipose tissue could be a new option for obesity control.

(−)-Epigallocatechin-3-gallate (EGCG) is the most abundant and active component of green tea. It has been reported to have chemopreventive and chemotherapeutic properties for obesity and obesity-related chronic diseases [13,14]. The previous studies focus on EGCG’s antioxidant and anti-inflammatory properties. Several studies demonstrated that EGCG inhibited tumor growth via anti-angiogenesis, in addition to inhibiting tumor cell proliferation and inducing apoptosis [15,16,17]. In our study, we hypothesized that EGCG could inhibit the growth and expansion of adipocytes via anti-angiogenesis. To determine this, we evaluated the effects of EGCG on adipogenesis and angiogenesis in 3T3-L1 cells and human umbilical vein endothelial cells (HUVECs).

## 2. Materials and Methods

### 2.1. 3T3-L1 Cell Culture and Differentiation

3T3-L1 preadipocyte cells from the American Type Culture Collection (ATCC-CL-173, Manassas, VA, USA) were cultured at 37 °C in a humidified atmosphere of 5% CO_2_. Cells were cultured in high-glucose Dulbecco’s Modified Eagle Medium (DMEM High-glucose, Hyclone, Shanghai, China) that contained 10% FBS (Gibco BRL Co.Ltd., Grand Island, NY, USA), 50 U/mL penicillin and 50 mg/mL streptomycin (Gibco BRL Co.Ltd., Grand Island, NY, USA). Two days after confluent (day 0), the 3T3-L1 cells were incubated in a differentiation medium containing 0.5 mM 3-iso-butyl-1-methylxanthine (Sigma–Aldrich, St. Louis, MO, USA), 1 µM dexamethasone (Sigma–Aldrich, St. Louis, MO, USA) and 10 µg/mL insulin (Sigma–Aldrich, St. Louis, MO, USA) in DMEM with 10% fetal bovine serum. After 48 h of stimulation, cells were cultured with DMEM containing 10 µg/mL insulin for 2 days and standard culture media for 4 days. The total duration of differentiation is 8 days. EGCG (Teavigo, DSM Nutritional Products Ltd., Basel, Switzerland) was dissolved in phosphate buffered saline (PBS) and added to the cell culture medium at concentrations of 0–25 µg/mL on day 0 [18,19]. The culture media with or without EGCG was changed every 2 days [20].

### 2.2. Monitoring of Cell Growth Using Real Time Cell Analysis (RTCA)

We used the xCELLigence RTCA DP instrument (Roche Diagnostics, Basel, Switzerland and ACEA Bioscience, San Diego, CA, USA) to perform real-time monitoring of cell survival according to the manufacturer’s instructions [21]. The background impedance signal was measured using 100 µL cell culture media per well. The final volume in a single well was adjusted to 200 µL by adding an additional 100 µL of media containing 6000 3T3-L1 fibroblasts (6000 cells/well). Impedance was routinely recorded in 15 min intervals once plated. After adherence, different concentrations of EGCG were added to the cell culture media. All incubations were performed in 200 µL total volume. For each concentration, three replicates on 16-well E-plates were performed. EGCG effects were analyzed at several different concentrations (0, 0.05, 0.1, 0.5, 1, 5, 10, 25, 50 µg/mL). To compare the results obtained from each wells, the normalized cell index (CI) was used. It was calculated as the CI at a given time point (CI_time x_) divided by the CI at the selected normalization time point (CI_norm time_) as follows: CI_(normalized)_ = CI_time x_/CI_norm time_ (termed here “normalized cell index”) [22]. The normalization time point selected was the time point prior to EGCG addition. The normalized cell index for all wells was 1 at the normalization time point. This normalized cell index was used to craft the graphical results representation.

### 2.3. Oil Red O Staining

3T3-L1 cells (4 × 10^4^ cells/well) were cultured in 96-well plates. After confluent, 3T3-L1 cells were treated with differentiation medium and different concentrations of EGCG (0, 5, 10, and 25 µg/mL) for 8 days. Culture media was removed, and cells were washed twice with PBS. Cells were then fixed using 4% paraformaldehyde for 1 h at room temperature. After fixation, the cells were washed with PBS three times and stained with 0.5% Oil red O (in 60% isopropanol) for 30 min at room temperature. After washing three times with PBS, the cells were inspected using an optical microscope. In addition to this gross evaluation, the dye was eluted for 10 min with 100% isopropanol. The concentration of the eluted dye was then determined by measuring absorbance (optical density (OD) 510 nm).

### 2.4. Immunofluorescence Staining

3T3-L1 cells (5 × 10^5^ cells/well) were cultured in 12-well plates. After confluent, 3T3-L1 cells treated with differentiation medium and different concentrations of EGCG (0, 5, 10, and 25 µg/mL) for 8 days. Then, 3T3-L1 cells were fixed with 4% paraformaldehyde for 15 min at room temperature and permeabilized with 0.2% Triton X-100 for 20 min at room temperature. Cells were washed 3× with 300 µL PBS and incubated with a primary antibody against VEGF (mouse anti-VEGF, Santa Cruz, Cambridge, UK, dilution 1:200) for 2 h in a humidified chamber. After three further washes with PBS, cells were incubated with Alexa Fluor^®^488 labeled anti-mouse antibody (Sigma–Aldrich, St. Louis, MO, USA) for 1 h. Subsequently, cells were washed three times with PBS. Cell nuclei were counterstained with 4′,6-diamidino-2-phenylindole (DAPI). Then, cells were washed with PBS and viewed using a fluorescence microscope.

### 2.5. Enzyme-Linked Immunosorbent Assay (ELISA)

3T3-L1 cells (5 × 10^5^ cells/well) were cultured in 12-well plates. The VEGF concentration of the conditioned media derived from 3T3-L1 cells treated with or without EGCG was measured using a VEGF enzyme-linked immunosorbent kit (R&D Systems, Minneapolis, MN, USA). 50 µL of Standard, Control and samples were added to each well, and then incubate for 2 h at room temperature. The assay procedure followed the manufacturer’s protocols. The VEGF concentration of each well was determined by measuring absorbance (OD 450 nm).

### 2.6. Protein Extraction and Western Blotting

3T3-L1 cells (5 × 10^5^ cells/well) were cultured in 12-well plates. After confluent, 3T3-L1 cells treated with differentiation medium and different concentrations of EGCG for 8 days. Then, cells were rinsed twice with ice-cold PBS, treated with Radio Immunoprecipitation Assay (RIPA) lysis buffer (Cell Signaling Technology, Inc., Danvers, MA, USA) and scraped. Next, cell lysates were centrifuged at 12,000× *g* for 10 min at 4 °C, and the supernatant was collected and stored at −80 °C. The protein concentrations of 3T3-L1 cells were measured using the bicinchoninic acid (BCA) method (Pierce Biotechnology, Inc., Rockford, IL, USA). An aliquot of 50 µg of supernatant protein was separated by 12% SDS-PAGE with 2× gel-loading buffer (100 mM Tris-HCl (pH 6.8), 4% SDS, 20% glycerol, 0.2% bromophenol blue, and 10% β-mercaptoethanol) and then blotted onto Immobilon-NC transfer membranes (Millipore, Bedford, MA, USA). Western blotting was performed using antibodies against PPARγ and C/EBPα (Cell Signaling Technology, Inc., Danvers, MA, USA) following the manufacturer’s protocols. The antibody for the internal control, α-tubulin, was purchased from Proteintech Group, Inc. (Chicago, IL, USA).

### 2.7. Real-Time Reverse Transcriptase–Polymerase Chain Reaction

3T3-L1 cells (5 × 10^5^ cells/well) were cultured in 12-well plates and were treated with or without EGCG. Total ribonucleic acid (RNA) was extracted with TRIzol reagent (Life Technologies, Inc., Grand Island, NY, USA) according to the manufacturer’s instructions. The quality and quantity of total RNA were determined by spectrophotometry (absorbance 260/280 nm). The total RNA samples (2 µg) were converted into complementary deoxyribonucleic acid (cDNA) by reverse transcription using the GoScript™ Reverse Transcription System (Promega, Madison, WI, USA). Briefly, the reaction was performed in a final volume of 20 µL, which included reaction buffer, PCR Nucleotide mix, random primers, MgCl_2_, GoScript™ Reverse Transcriptase, RNase inhibitor and RNA. The reaction mixtures were heated at 25 °C for 5 min, 42 °C for 60 min and 70 °C for 15 min. Real-time PCR was performed using the 7000 Real-Time PCR System (Applied Biosystems, Foster, CA, USA). Each well was brought to a final volume of 20 µL, which included GoTaq^®^ qPCR Master Mix (Promega, Madison, WI, USA), an optimized concentration of each primer and 2 µL of cDNA. The reaction mixtures were heated at 95 °C for 15 min to activate the enzyme and then subjected to 40 cycles of melting at 95 °C for 15 s and annealing/extension at 60 °C for 1 min. The mRNA levels of all genes were normalized using β-actin as an internal control. The following primers were used in the PCR reactions: VEGF-A forward, 5′-GAAAGGCTTCAGTGTGG-3′ and reverse, 5′-CAGGAATGGGTTTGTCG-3′; PPARγ forward, 5′-TCACAATGCCATCAGGT-3′ and reverse, 5′-GCGGGAAGGACTTTATGTA-3′; C/EBPα forward, 5′-GCCCCTCAGTCCCTGTCTTTA-3′ and reverse, 5′-AGCCCTCCACCTCCCTGTAG-3′; β-actin forward, 5′-CCTCTATGCCAACACAGT-3′ and reverse, 5′-AGCCACCAATCCACACAG 3′.

### 2.8. Co-Culture of HUVEC and 3T3-L1 Cells

HUVECs (ALLCELLS, Shanghai, China) were seeded (6000 cells/well) into E-plates. The cell growth curves were recorded at 15 min intervals on the xCELLigence System in real time. 3T3-L1 preadipocytes were adjusted to 1.25 × 10^3^, 2.5 × 10^3^ and 5 × 10^3^ in 50 µL of DMEM, and 3T3-L1 adipocytes were adjusted to 1.25 × 10^3^ in 50 µL of DMEM. Then, cells were added to the insert in the CCD receiver containing 130 µL DMEM. After adherence, the insert containing 3T3-L1 cells was taken out of the CCD receiver and was put into the E-plates. Then, E-plates were placed back in the xCELLigence station, and the xCELLigence software program was continued so that impedance readings were taken every 15 min. Finally, the results were normalized at the end of the assay. HUVEC media was not supplemented with VEGF.

### 2.9. Tube Formation Assay for in Vitro Angiogenesis

For the tube formation assay, HUVECs were incubated with VEGF-free MCDB131 medium (ALLCELLS, Shanghai, China) for 24 h. Matrigel was thawed at 4 °C in an ice-water bath. Matrigel was then carefully added to a pre-chilled 96-well plate (50 µL per well) using a cold pipette and allowed to polymerize for 1 h at 37 °C. After polymerization, HUVECs (3 × 10^3^ cells/well) were incubated for 24 h at 37 °C with 50 µL of MCDB131 and 150 µL of the conditioned media derived from post-differentiated 3T3-L1 cells in the presence or absence of anti-mouse VEGF-neutralizing antibody. Three different phase-contrast microscopic low-power fields (×40) per well were photographed. The total length of capillary tubes in each photograph was measured using a scale ruler.

### 2.10. Statistical Analyses

Data were presented as means ± standard deviations. Comparison of multiple treatment conditions with control were conducted by using one-way analysis of variance (ANOVA) followed by Dunnett’s test (if equal variance is met among groups) or Dunnett’s T3 test (if equal variance is not met among groups). All analyses were performed by using SPSS for windows (release 19.0). A value of *p* ≤ 0.05 was considered statistically significant. In addition, all tests are two-sided.

## 3. Results

### 3.1. Inhibitory Effects of EGCG on 3T3-L1 Preadipocyte Growth, Differentiation and Fat Accumulation

**Figure 1:**
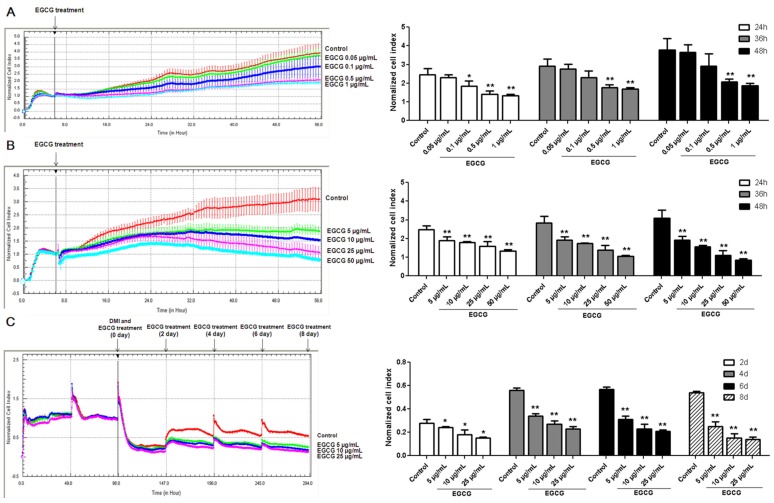
Effects of (−)-Epigallocatechin-3-gallate (EGCG) on the proliferation and differentiation in 3T3-L1 cells. The effects of EGCG at physiologically attainable concentrations (**A**) and higher concentrations (**B**) on the proliferation of 3T3-L1 preadipocytes. 3T3-L1 preadipocytes were seeded into E-plates at equal densities of 6000 cells/well. For each concentration, three replicates on a 16-well E-plate were performed. Impedance at well bottoms was measured every 15 min for 48 h and the impedance signal was analyzed by normalizing data of each single well to the first measurement after EGCG treatment; (**C**) The effects of EGCG on differentiation of 3T3-L1 cells. 3T3-L1 preadipocytes were seeded into E-plates at equal densities of 10,000 cells/well. The medium with or without EGCG was changed every 2 days. Changes in impedance were normalized to the time of differentiation medium (DMI) addition and were given as a dimensionless normalized cell index (CI) values. Normalized cell index reflects the cell density. Values are means ± standard deviation (SD), *n* = 3. * *p* < 0.05, ** *p* < 0.01 *vs.* the control.

To explore the effects of EGCG on preadipocyte growth, 3T3-L1 preadipocytes were treated with physiologically attainable concentrations (0.05, 0.1, 0.5 and 1 µg/mL) and higher concentrations of EGCG (5, 10, 25 and 50 µg/mL) for 2 days after seeding. The media with or without EGCG were not replaced during the incubation time. Compared with that in the control group, the normalized cell index in the groups using 0.5 µg/mL and 1 µg/mL EGCG, which reflects cell density, were significantly decreased (Figure 1A). As shown in Figure 1B, the normalized cell index in the groups using higher concentrations of EGCG was significantly lower than that in the control group.

**Figure 2:**
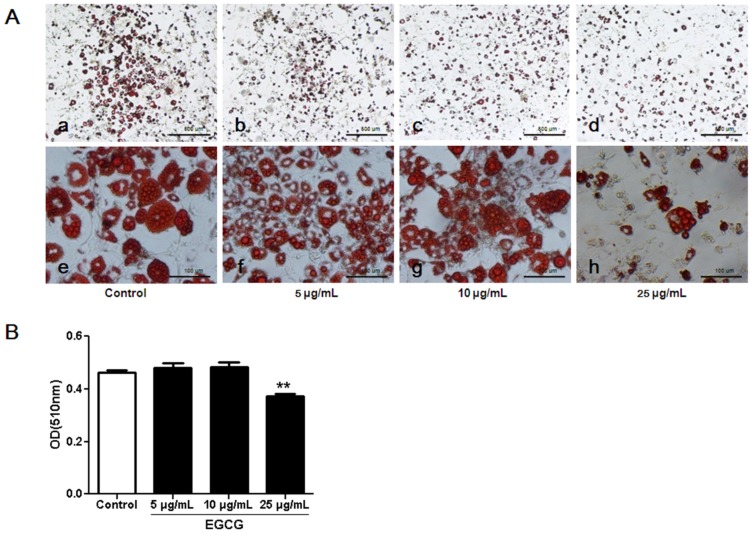
The effects of (−)-Epigallocatechin-3-gallate (EGCG) on the inhibition of fat accumulation in 3T3-L1 adipocytes. (**A**) The 3T3-L1 adipocytes were treated with different concentrations of EGCG and lipid droplets were stained with Oil red O stain. The cells were photographed at magnification ×40 (**a**–**d**) and ×200 (**e**–**h**). The experiment was performed three times in triplicate for each concentration. Representative images are shown; (**B**) The oil red O stained cells were treated with isopropanol to extract the dye, and the concentration of the eluted dye was determined by absorbance at optical density(OD) 510 nm. Error bars represent standard deviation of the mean, *n* = 3. ** *p* < 0.01 *vs.* the control.

Following incubation of 3T3-L1 preadipocytes with differentiation medium (DMI) containing different concentrations of EGCG (0, 5, 10 and 25 µg/mL) [20], we examined the normalized cell index during adipocyte differentiation. On the other hand, we also performed Oil Red O staining to observe whether EGCG altered fat accumulation in 3T3-L1 adipocytes. As shown in Figure 1C, the normalized cell index dropped quickly when the cell shapes were changed after adding DMI. Additionally, we found that the normalized cell index was significantly decreased when 3T3-L1 cells were incubated with 5, 10, and 25 μg/mL of EGCG as compared to the control group. A significant inhibition of preadipocyte differentiation to mature adipocytes was observed when EGCG concentration was 25 µg/mL as evidenced by less fat accumulation in the cells (Figure 2).

### 3.2. Regulation of Transcription Factors in 3T3-L1 Adipocytes by EGCG

**Figure 3:**
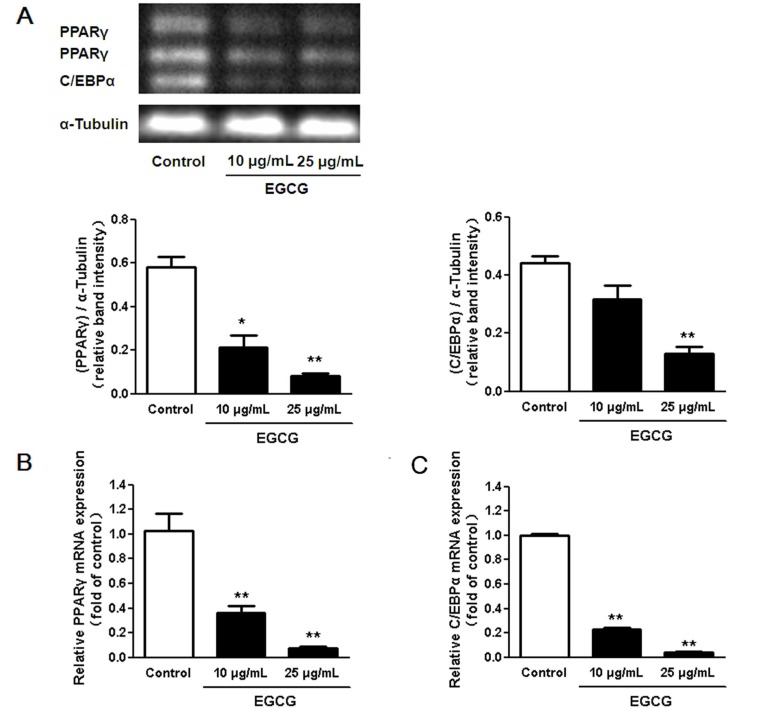
Effects of (−)-Epigallocatechin-3-gallate (EGCG) on the expression of adipogenic transcription factors during adipogenesis. (**A**) EGCG decreased both C/EBPα and Peroxisome proliferator-activated receptor γ (PPARγ) protein expression levels in a dose-dependent manner. Changes in expression of PPARγ (**B**) and C/EBPα (**C**) in differentiated 3T3-L1 adipocytes treated with EGCG as analyzed by quantitative real-time polymerase chain reaction (PCR). Results were normalized to the expression of α-tubulin and are expressed as relative mRNA level compared with the average expression in cells incubated without EGCG. The error bars represent the standard deviation (SD) (*n* = 3); * *p* < 0.05, ** *p* < 0.01 *vs.* the control.

Since we found that the lipid accumulation was significantly decreased when 3T3-L1 cells were incubated with 25 μg/mL of EGCG compared to the control group, 10–25 μg/mL of EGCG were used to examine the effect of EGCG on regulating the relevant transcript factors. We measured the expression of PPARγ and C/EBPα to determine whether EGCG treatment altered adipocyte marker gene expression in 3T3-L1 adipocytes. As shown in Figure 3, the expression of PPARγ and C/EBPα was significantly down regulated after 10 to 25 μg/mL of EGCG treatment throughout the entire differentiation period.

### 3.3. Inhibitory Effects of EGCG on VEGF Expression in both 3T3-L1 Preadipocytes and Adipocytes

**Figure 4:**
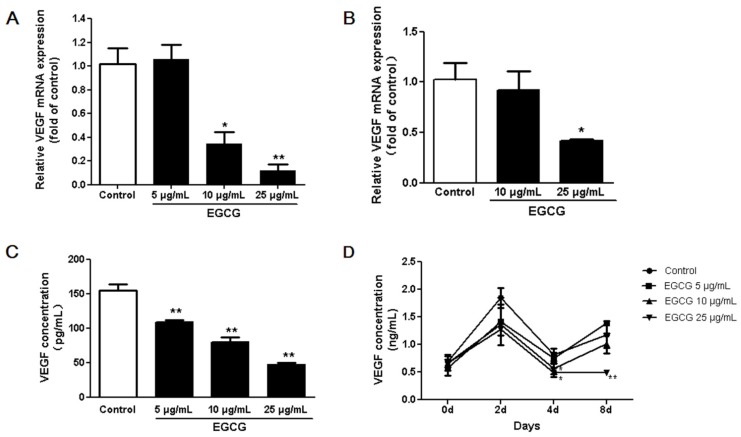
The effects of (−)-Epigallocatechin-3-gallate (EGCG) on vascular endothelial growth factor (VEGF) expression in 3T3-L1 preadipocytes and adipocytes. VEGF mRNA levels of 3T3-L1 preadipocytes (**A**) and adipocytes (**B**) as quantified using 7000 Real-Time PCR System (Applied Biosystems); (**C**) VEGF concentrations in 3T3-L1 preadipocyte conditioned media which was treated with or without EGCG for 48 h as analyzed by enzyme linked immunosorbent assay (ELISA); (**D**) VEGF concentrations in the conditioned media from 3T3-L1 cells treated with or without EGCG during adipocyte differentiation as measured by ELISA. Data are expressed as a percentage of the control (non-treated cells). Values are expressed as mean ± standard deviation (SD), *n* = 3. * *p* < 0.05, ** *p* < 0.01 *vs.* the control.

**Figure 5:**
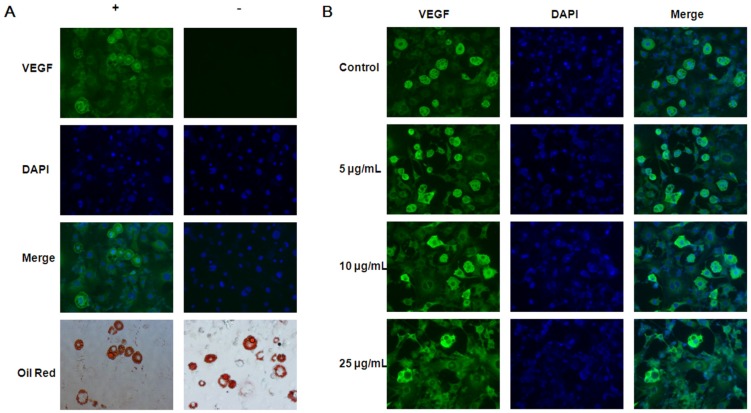
The (−)-Epigallocatechin-3-gallate (EGCG) regulation of vascular endothelial growth factor (VEGF). (**A**) 3T3-L1 adipocytes that were fixed, permeabilized, stained with or without VEGF (green). Cell nuclei were stained with DAPI (blue). Lipid droplets were stained with Oil Red O. The results were observed by fluorescence microscope; (**B**) Cells treated with EGCG 0–25 μg/mL during differentiation, fixed, permeabilized and double stained with VEGF and anti‑mouse 448 secondary antibody (green). Cell nuclei were stained with DAPI (blue) (magnification: ×200). Results were representative of three independent experiments, *n* = 3.

According to its effect on the proliferation and differentiation of 3T3-L1 cells, we used 5–25 μg/mL of EGCG to explore the effect of EGCG on angiogenesis. We tested the effects of EGCG on VEGF expression in adipocytes with four sets of experiments. First, real time PCR results showed that EGCG decreased VEGF gene expression in both 3T3-L1 preadipocytes and adipocytes (Figure 4A,B). Second, our experiment showed that VEGF protein secretion by 3T3-L1 preadipocytes was significantly inhibited by EGCG at all concentrations 5–25 µg/mL (Figure 4C). Third, the ELISA assay showed that during differentiation the VEGF concentrations in conditioned media dramatically increased after adding the induction media. At the end of differentiation, the VEGF concentrations in conditioned media of matured adipocytes were also higher than that of preadipocytes. VEGF concentrations in the culture media of 3T3-L1 adipocytes were decreased in EGCG treated cells (Figure 4D). Finally, we verified that the fluorescence intensity of VEGF in adipocytes was higher than in preadipocytes by immunofluorescence staining (Figure 5).

### 3.4. Coculture of 3T3-L1 Cells with HUVECs

**Figure 6:**
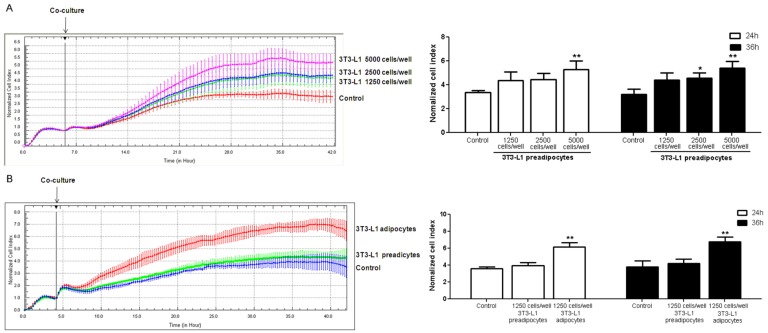
Real-time monitoring of human umbilical vein endothelial cells (HUVECs) cultured with 3T3-L1 cells. HUVECs were seeded into E-plates at equal densities of 6000 cells/well. (**A**) 3T3-L1 preadipocytes seeded at different densities into the insert; (**B**) 3T3-L1 preadipocytes and 3T3-L1 adipocytes seeded into E-plates at equal densities of 1250 cells/well. There were no cells in the insert of control group. After adherence, the insert was added into the E-plates containing HUVECs. Changes in cell index were normalized to the time of co-culture. Experiments were performed in triplicates. The error bars represent the standard deviation (SD) (*n* = 3); * *p* < 0.05, ** *p* < 0.01 *vs.* the control.

In order to test the role of adipocytes in angiogenesis, coculture of 3T3-L1 cells and HUVECs was performed. The results showed that cell density of HUVECs rose with the increase of 3T3-L1 preadipocytes (Figure 6A). Compared with preadipocytes, mature adipocytes further increased the cell density of HUVECs (Figure 6B).

### 3.5. Tube Formation in HUVECs Treated with Conditioned Media from 3T3-L1 Cells

**Figure 7:**
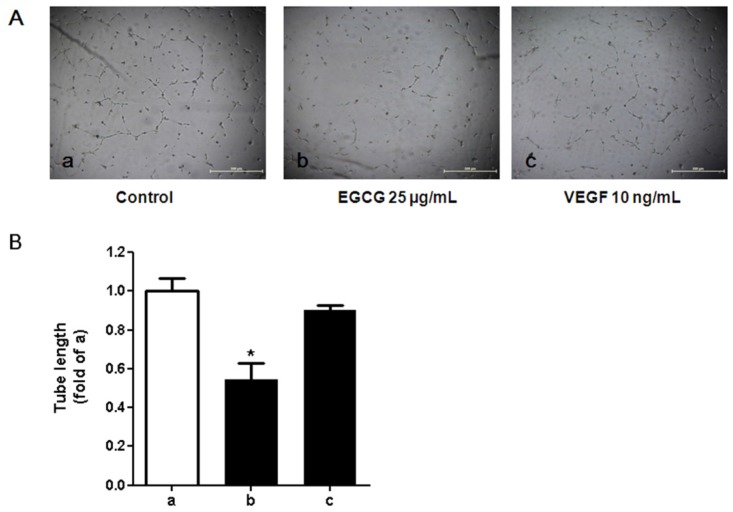
Effects of the conditioned media cultured with 3T3-L1 adipocytes on tube formation activity in human umbilical vein endothelial cells (HUVECs). (**A**) Light microscopic images of HUVEC tube formation in various conditions. HUVECs were seeded on Growth Factor Reduced BD Matrigel matrix (BD Biosciences, San Jose, CA, USA) with conditioned medium derived from 3T3-L1 adipocytes in the presence or absence of (−)-Epigallocatechin-3-gallate (EGCG) or recombinant vascular endothelial growth factor (VEGF) protein. After 24 h of stimulation, phase-contrast microscopic low-power fields (×40) were photographed. **a**: with conditioned media from 3T3-L1 adipocytes in the absence of EGCG; **b**: with conditioned media from 3T3-L1 adipocytes in the presence of EGCG (25 µg/mL); **c**: stimulated with recombinant VEGF (10 ng/mL); (**B**) Tube length of HUVECs stimulated with conditioned medium prepared from 3T3-L1 adipocytes. Total length of capillary tubes formed by HUVECs in 3 different photographs per well was measured using a scale ruler. Bar a: with conditioned media from 3T3-L1 adipocytes in absence of EGCG; bar b: with the conditioned media from 3T3-L1 adipocytes in presence of EGCG (25 µg/mL); bar c: stimulated with recombinant VEGF (10 ng/mL). The error bars represent the standard deviation (SD) (*n* = 3). * *p* < 0.05 *vs.* the control (a).

Since 25μg/mL EGCG significantly decreased VEGF concentrations and suppressed VEGF mRNA expression in the culture of preadipocytes and adipocytes, the conditioned media from cultured 3T3-L1 adipocytes which was treated with 25 μg/mL EGCG was then used to perform the tube formation assay for *in vitro* angiogenesis. We observed that the conditioned media, which contained the secretions from 3T3-L1 adipocytes (e.g., VEGF was verified), enhanced tube formation in HUVECs (Figure 7). The conditioned media derived from adipocytes treated with EGCG inversely affected the tube formation.

## 4. Discussion

Previous studies have shown that the growth and expansion of adipose tissue is accompanied by vascularization via angiogenesis [1]. VEGF, one of the adipocytokines, contributes most of the angiogenic activity of adipose tissue [23]. Adipose tissue-specific VEGF knockout mice that are fed a high fat diet have increased adipocyte apoptosis [8]. In our study, we observed that mature adipocytes secreted more VEGF when compared with preadipocytes. The results suggest that by secreting more VEGF mature adipocytes could induce more angiogenesis compared to preadipocytes. Expansion of adipocyte size in adipose tissue increases the intercapillary distance. This phenomenon leads to a relatively hypoxic environment that induces VEGF expression and other reactions like inflammation [24]. EGCG is one of the natural antioxidants that can tolerate hypoxia [25,26]. Our results showed that EGCG inhibited VEGF expression in both preadipocytes and adipocytes. This interesting finding indicates that EGCG may inhibit the excessive proliferation and differentiation of fat cells through the suppression of both angiogenesis and adipogenesis. On the other hand, when using HUVECs, we also found that adipocytes could stimulate the proliferation of vascular endothelial cells by secreting VEGF. Furthermore, this process could be suppressed by EGCG. Thus, we report here that EGCG can inhibit angiogenesis in adipose tissue.

In the HUVEC experiment, we did not measure the adipokine composition of the adipocyte conditioned media before addition to the HUVECs. However, there should be angiogenic factors, various pro-inflammatory cytokines and growth factors in the media as previously reported [23,27]. Because of this, we conclude that VEGF may serve as a crucial factor for the growth of HUVECs. Nevertheless, mechanisms other than the VEGF pathway by which EGCG could affect angiogenesis should also be considered. For example, it has been reported that EGCG has anti-inflammatory effects, through its modulation of pro-inflammatory cytokine tumor necrosis factor-α (TNF-α) and interleukin-6 (IL-6) [28]. It is possible that EGCG improves pathological neovascularization in part by its anti-inflammatory effects. Confirming this possibility, however, will need further study.

Of note, EGCG is not stable in solution and can be autooxidized at neural pH and produces some metabolites, e.g., dimers [29]. Indeed, EGCG may be metabolically activated to form more potent and effective bioactive compounds. Although any potential impact of EGCG intake may reflect the combined effects of EGCG and its metabolites, we aimed to examine the effect of a certain amount intake of EGCG on adipogenesis and angiogenesis in 3T3-L1 adipocytes in the present study.

There are few published data reported the concentration of EGCG in adipose tissue after its intake, which should be further studied. Previous studies have showed that the concentration of EGCG in human plasmais < 1 μM after consuming one cup of green tea [30,31]. However, it is slowly removed from the body and may accumulate in tissues over time to produce cellular concentrations, therefore higher plasma concentrations of EGCG could be achieved when it is regularly consumed [32]. In order to explain the biological response of adipocytesto EGCG, physiologically attainable (0.05, 0.1, 0.5, 1 μg/mL) and higher concentrations (5, 10, 25, 50 μg/mL) of EGCG were used in this study. We used an advanced instrument, the xCELLigence RTCA DP system, to study the growth and differentiation of 3T3-L1 preadipocytes. This new instrument provides an unhindered real time view of whole cell biology and offers other benefits over traditional end point assays like the MTT assays. The practicality of this system has been shown in recent obesity studies and is a viable primary method of screening compounds for adipogenic factors [33]. Using this new method, we verified that EGCG can suppress adipogenesis by inhibiting the differentiation of preadipocytes and the proliferation of adipocytes. These findings are consistent with earlier studies [34]. Moreover, we observed that EGCG at 25 μg/mL (corresponding to 50 μM) decreased fat accumulation by decreasing cell numbers. Results from this study and previous studies [35,36,37] indicate that EGCG at concentrations of ≥50 μM may induce apoptosis and inhibit differentiation of preadipocytes. Due to the limited numbers of E-plate wells, we cannot cover all EGCG concentrations from 0 to 50 µg/mL in one plate by design. Therefore we cannot examine dose-response relationship between EGCG and normalized cell index, which should be considered in the further study.

The mechanism through which EGCG inhibits adipocyte to produce VEGF has been unclear thus far. One possible mechanism is that, as mentioned above, EGCG can inhibit angiogenesis by ameliorating hypoxia that is induced by accumulation and expansion of the adipose tissue. The other possible mechanism is that EGCG has an inhibitory effect on PPARγ. PPARγ and C/EBPα are the main transcription factors in adipogenesis and lipogenesis. Down-regulation of PPARγ and C/EBPα expression by EGCG would be expected to be inhibitory to lipid deposition and adipocyte differentiation. In particular, PPARγ has a net pro-angiogenic role in the context of adipose tissue [38]. However, the detailed mechanism underlying the down-regulation of PPARγ by EGCG has not been fully elucidated. Inhibition of PPARγ function in pre-adipocytes prevents not only the differentiation of adipocytes, but also the formation of vasculature *in vivo* [38]. This is likely due to the secretion of pro-angiogenic factors from adipocytes in response to PPARγ activation [39]. In this study, we found that EGCG down-regulated the level of PPARγ in adipocytes. Therefore, it seems reasonable that EGCG may play a role in angiogenesis through the PPARγ pathway. However, both of the above mechanisms need further studies to clarify.

## 5. Conclusions

The findings from this study suggested that EGCG could inhibit the growth and expansion of adipocytes via down regulation of VEGF expression and secretion *in vitro*. However, the effect of EGCG on adipose tissue’s angiogenesis should be further investigated *in vivo*.
